# Supplementary material for: Cost stickiness, absorbed slack and enterprise risks: Evidence from China
Source: PLoS One. 2024 Dec 20;19(12):e0315675. doi: 10.1371/journal.pone.0315675 (PMC11661607; doi:10.1371/journal.pone.0315675)
Supplement: Attachment — Submitted filename: Rebuttal Letter.docx [file pone.0315675.s001.docx]

**Rebuttal Letter**

**Response to reviewers**

Dear reviewer,

Thank you very much for your time in reviewing the manuscript and your encouraging comments on the merits. We also appreciate your clear and detailed feedback and hope the explanation has fully addressed your concerns. In the remainder of this letter, we discuss your comments individually and our corresponding responses.

To facilitate this discussion, we first retype your comments in *italic font* and then present our responses to the comments.

**Comment 1:**

*Lack of Clarity and Focus in Abstract and Introduction:
• The abstract does not clearly define the research problem or provide a concise summary of methodology, findings, and conclusions.
• The introduction lacks a strong global context of the issue before delving into specific country implications. It should incorporate a compelling hook and a clear thesis statement to engage readers effectively.*

**Response 1:**

Your suggestion is very relevant. As a response, We have rewritten the abstract section of the paper to more clearly define the problem to be studied and to provide a more concise summary of the research methodology, findings, and conclusions. At the same time, we have rewritten the introduction section so that the director can be more compelling and have a clearer statement of the topic to effectively engage the reader.

**Comment 2:**

*Methodological Complexity and Presentation Issues:*

*• The methodology section needs simplification to enhance readability. Complex formulas and data processes should be explained more clearly for better understanding.*

*• The paper lacks a robust justification for the data source used and does not sufficiently discuss its validity and authority, which raises concerns about the reliability of the research.*

**Response 2:**

Thanks for the detailed references.We have optimized the research methods section so that the readers can read it more clearly. As to the formulas, we have given more explanations, such as formula (6) and the O-score in section 4.5. Data processing has also been optimized so that the readers can better understand it.

As for the data sources, we have made special explanations in section 3.1 to elaborate the validity and authority of the data and to ensure the reliability of the research. We obtained all the data from the CSMAR(China Stock Market & Accounting Research) database. This database is a comprehensive financial database widely recognized for its extensive coverage of Chinese financial markets. It encompasses a broad spectrum of data including stock market information, accounting data, economic indicators, and corporate governance details. The database is known for its rigorous data collection and verification processes, ensuring high data quality and reliability. Its effectiveness is underscored by its widespread use in academic research and professional analyses, making it a trusted source for financial data in China. The authority of CSMAR is further bolstered by its alignment with international standards and its commitment to transparency and accuracy, solidifying its position as a leading database in the field.

**Comment 3:**

*Insufficient Literature Review and Theoretical Framework:
• The literature review is not comprehensive enough, lacking detailed supporting theories and references to strengthen the credibility and depth of the paper.
• The theoretical analysis is also missing, which is essential for providing a strong foundation and context for the research.*

**Response 3:**

It is a vital part to relect the research value. Following your suggestion, we have rewritten section 1: “Introduction”, section 2.1: “Cost Stickiness and enterprise risks” and section 2.2: “Mediating role of absorbed slack” to strengthen the credibility and depth of the paper with more literature review, detailed supporting theories and references. In addition, Fig. 1. “Theoretical framework of the hypotheses” is added.

**Comment4:**

*Overabundance of Statistical Tests and Ambiguity in Findings:
• The paper includes an excessive number of statistical tests, which detracts from the clarity and effectiveness of the analysis. Reviewers suggest focusing on essential tests (e.g., variable loadings, R2) to maintain clarity and impact.
• Findings are presented ambiguously, making it difficult to interpret the results and limiting the generalizability of the research to broader contexts.*

**Response 4:**

As you mentioned, we reanalyzed the empirical section to clarify the results and assure validity. At the same time, we pay more attention to the basic tests such as R^2^ as indicated by reviewers to maintain clarity and impact of the results.

We have rewritten the results part to better explain the research results. In addition, we have increased the length of the paper to explain the findings more clearly to ensure that the study can be generalized in a broader context. Please see section5: “Conclusions and Suggestions”.

In summary, we would like to thank you for all your time involved and for this excellent opportunity for us to improve the manuscript. We hope you will find this revised version satisfactory.

Sincerely,

Binhua Qian

**Response to the editor**

Dear editor,

Thanks for your reply mail about our submission PONE-D-24-03783. We appreciate the detailed and constructive comments provided by you and the reviewers. We are submitting the revised manuscript entitled “Cost Stickiness, Absorbed Slack and Enterprise Risks: Evidence from China” for consideration for publication in PLOS ONE.

In the revision, we have performed all the suggested data and analyses, and fully addressed the comments made by the reviewers and editor. The finished new parts are as following:

1. About “Lack of Clarity and Focus in Abstract and Introduction”. We have revised the abstract and introduction section of this manuscript to be more clear. Also, We try to use a more specific topic statement to effectively engage the reader.
2. About “Methodological Complexity and Presentation Issues”. We have added explanations to the formulas and data processing to make the manuscript more clear. As to the data source, we use a paragraph to explain the CSMAR database in order to prove the authority of the data source.
3. About “Insufficient Literature Review and Theoretical Framework”. We have reorganized the literature review part and the theoretical framework part, added theoretical expositions on introduction section and subsection 2.2.
4. About “Overabundance of Statistical Tests and Ambiguity in Findings”. We have checked the statistical test processes a second time, and reorganized the “conclusions and suggestions” section, so as to explain the results and findings more clearly.

We hope this revised manuscript has addressed your concerns, and look forward to hearing from you.

Sincerely,

Binhua Qian

Professor in Economy and Management

School of Economy and Management, Zhejiang Business Technology Institute

1988 Jichang Road, Haishu District, Ningbo, China

E-mail: herrgeld@126.com
